# In Silico Screening for Novel Leucine Aminopeptidase Inhibitors with 3,4-Dihydroisoquinoline Scaffold

**DOI:** 10.3390/molecules25071753

**Published:** 2020-04-10

**Authors:** Joanna Ziemska, Jolanta Solecka, Małgorzata Jarończyk

**Affiliations:** 1 National Institute of Public Health–National Institute of Hygiene, Chocimska 24, 00-791 Warsaw, Poland; jsolecka@pzh.gov.pl; 2 National Medicines Institute, Chełmska 30/34, 00-725 Warsaw, Poland; m.jaronczyk@nil.gov.pl

**Keywords:** leucine aminopeptidase inhibitor, 3,4-dihydroisoquinoline, molecular docking, structure-based drug design, 3D-QSAR, drug-likeness

## Abstract

Cancers are the leading cause of deaths worldwide. In 2018, an estimated 18.1 million new cancer cases and 9.6 million cancer-related deaths occurred globally. Several previous studies have shown that the enzyme, leucine aminopeptidase is involved in pathological conditions such as cancer. On the basis of the knowledge that isoquinoline alkaloids have antiproliferative activity and inhibitory activity towards leucine aminopeptidase, the present study was conducted a study which involved database search, virtual screening, and design of new potential leucine aminopeptidase inhibitors with a scaffold based on 3,4-dihydroisoquinoline. These compounds were then filtered through Lipinski’s “rule of five,” and 25 081 of them were then subjected to molecular docking. Next, three-dimensional quantitative structure-activity relationship (3D-QSAR) study was performed for the selected group of compounds with the best binding score results. The developed model, calculated by leave-one-out method, showed acceptable predictive and descriptive capability as represented by standard statistical parameters r^2^ (0.997) and q^2^ (0.717). Further, 35 compounds were identified to have an excellent predictive reliability. Finally, nine selected compounds were evaluated for drug-likeness and different pharmacokinetics parameters such as absorption, distribution, metabolism, excretion, and toxicity. Our methodology suggested that compounds with 3,4-dihydroisoquinoline moiety were potentially active in inhibiting leucine aminopeptidase and could be used for further in-depth in vitro and in vivo studies.

## 1. Introduction

Cancer incidence and mortality are rapidly increasing, and cancer is presently the most important barrier to increase life expectancy worldwide. According to the World Cancer Report published by the World Health Organization in 2020, cancer is the first or second cause of premature death (i.e., in the age group of 30–69 years) in 134 of 183 countries, and it ranks third or fourth in additional 45 countries [1]. The most commonly diagnosed types of cancer in both genders are lung cancer, female breast cancer, colorectal cancer, prostate cancer, stomach cancer, and liver cancer. Lung cancer remains the leading cause of cancer incidence and mortality globally [2]. 

Many studies have shown that overexpression of the metalloenzyme leucine aminopeptidase (LAP) plays a role in the proliferation, migration, and invasion of tumor cells and in angiogenesis [3,4]. Leucine aminopeptidase 3 (LAP 3) was reported to be involved in the histological grade, lymph node metastasis, angiogenesis, proliferation, malignant development, and prognosis of endometrial cancer, ovarian cancer, esophageal cancer, liver cancer, and glioma [3,4,5,6,7]. 

Activities of various aminopeptidases, including alanyl, arginyl, cystinyl, glutamyl, aspartyl, and pyroglutamyl aminopeptidase, in breast cancer tissues was studied by Martinez et al. [8]. The activity of alanyl, arginyl, and cystinyl aminopeptidases was observed to be increased in breast cancer. Oxytocin inhibits the proliferation of human breast cancer cell lines and thus may play a role in preventing cancer. Both cystinyl and leucine aminopeptidases cleave oxytocin and may be involved in the development of breast cancer [8].

The process of introducing a novel drug in the market requires a huge investment in time and money. It was estimated that approximately 2.6 billion dollars are required to develop and introduce a drug in the market, and the cost has increased almost 150% in the last decade. However, the failure rate has also increased to nearly 90% [9]. Various computer-aided drug discovery (CADD) techniques are available that enable the development of new chemical entities. Structure-based drug design (SBDD) and the knowledge of 3D-structural data of targets enable the visualization of the binding process of ligands to targets and to predict the key binding pocket sites and affinity of ligands to their target macromolecules. Three-dimensional quantitative structure-activity relationship (3D-QSAR) analysis based on the nature of molecular interaction can provide affluence information about the exact molecular characteristics essential for biological activity and serve as a significant predictive tool predominantly for designing pharmaceuticals [10]. QSAR allows the quantification of the relationship between the structure of the ligand and its biological activity. It also helps in the optimization of the groups that modulate drug potency and the rationalization of drug components which leads to better activities; it can also be used as a screening tool [11].

The determination of the architecture of the active site of the macromolecule can provide clear information about the protein-ligand interaction phenomenon, post-docking dynamics, hydrogen bond formation, and free energies of the complex [12].

Bovine lens leucine aminopeptidase (blLAP) is a well-characterized M17 aminopeptidase with binuclear metal center containing two essential metal ions to selectively remove leucine residues from short peptides [13]. Its crystal structure is well defined (Protein Data Bank, PDB code: 1LAN), and it is often used in several theoretical studies. As observed by Drinkwater et al., the term “leucine aminopeptidase” encompasses a range of protein families, including M17 (hexameric with binuclear metal center), M1 (monomeric with a single metal center), and occasionally M18 (monomeric with a binuclear metal center) and M28 aminopeptidases (monomeric with a binuclear metal center), and enzymes that release not only leucine from peptides but also other amino acids such as cysteine, glycine, arginine, lysine, or proline [13].

**Figure 1:**
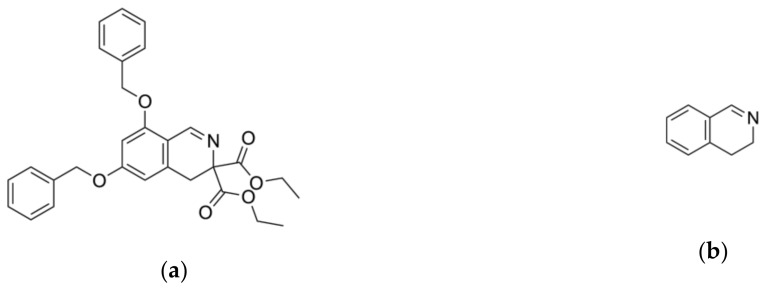
(**a**) chemical structure of diethyl 6,8-dibenzyloxy-3,4-dihydroisoquinoline-3,3-dicarboxylate and (**b**) chemical structure of the 3,4-dihydroisoquinoline scaffold.

In the previous studies, we discovered that a group of 3,4-dihydroisoquinoline (Figure 1b) derivatives exhibit LAP inhibitory activity [14]. We also proved that one of the studied compounds, diethyl 6,8-dibenzyloxy-3,4,-dihydroisoquinoline-3,3-dicarboxylate (Figure 1a), exhibited significant activity against microsomal LAP (IC_50_ = 16.5 µM) and promising antiproliferative activity on human cancer cell lines, including human promyelocytic leukemia cell line HL-60, human breast cancer cell line MCF-7, Burkitt’s lymphoma cell line Raji, camptothecin resistant CEM/C2 human T-cell leukemia cell line with mutated catalytic site of topoisomerase 1 and its parental cell line CCRF/CEM, LoVo colon cancer cell line, LoVo/Dx-variant cell line resistant to doxorubicin with multidrug cross-resistance, prostate cancer cell lines LNCaP and PC3, and urinary bladder cancer cell line HCV29T. Moreover, diethyl 6,8-dibenzyloxy-3,4,-dihydroisoquinoline-3,3-dicarboxylate showed a high selectivity index towards the studied cancer cell lines versus normal mammalian cells and exhibited a promising safety profile in toxicological studies. In molecular docking studies, the discovered compound exhibited hydrophobic and H-bonding interactions with amino acids residues essential for LAP inhibitory activity. Moreover, the compound’s influence on cell cycle and cell death was determined and the studies confirmed the role of diethyl 6,8-dibenzyloxy-3,4,-dihydroisoquinoline-3,3-dicarboxylate in the inhibition of cell cycle G1/S transition. 

Our findings regarding the activity of diethyl 6,8-dibenzyloxy-3,4,-dihydroisoquinoline-3,3-dicarboxylate were consistent with those of other authors such as Bermejo et al. [15]. These authors discovered isoquinolines with antitumor activity that target the G1 phase of the cell cycle. As G1 phase of the cell cycle is an important period and its malfunction is critical for tumorigenesis and tumor progression, the agents with the ability to arrest cells in the G1 phase can be highly promising new therapeutic agents against human cancers.

Several studies have reported phosphonic acid analogues of leucine as inhibitors of both cytosolic and microsomal aminopeptidases [16,17] and phosphonate and phosphonamidate inhibitors as excellent inhibitors of zinc metallopeptidases. Therefore, we performed theoretical studies, including 3D-QSAR on phosphonic/phosphinic acid-containing isoquinoline derivatives [18]. Our previous research provided essential information on the structural characteristics of the inhibitors and gave new insight into the discovery of LAP inhibitors. 

Considering the unique characteristics of diethyl 6,8-dibenzyloxy-3,4-dihydroisoquinoline-3,3-dicarboxylate and other 3,4-dihydroisoquinoline derivatives, we selected them as perfect candidates for further *in silico* studies. Here, modern drug discovery techniques were applied, such as database search, virtual screening (VS), ligand growing experiment, molecular docking, 3D-QSAR, and absorption, distribution, metabolism, excretion, and toxicity (ADMET), to develop novel potential LAP inhibitors.

## 2. Results and Discussion

### 2.1. Database Search and Library Establishment

Different compound databases such as ZINC [19], PubChem [20], and DrugBank [21] were searched for dihydroisoquinoline analogues. Then, by using Lipinski’s “rule of five” [22,23], molecules with less reasonable physicochemical parameters were discarded, leading to the selection of candidates with good drug-like properties: molecular weight (MW) <500 Da, number of hydrogen bond donors <5, number of hydrogen bond acceptors <10, log_10_ partition coefficient (logP) <5, and no more than one violation of the abovementioned criteria. A virtual library of approximately 11 585 compounds extracted from ZINC and 13 011 compounds extracted from PubChem was established. DrugBank database did not show any significant hits.

### 2.2. Ligand Growing Experiment

Independent from database search, the design of LAP inhibitors using the ligand growing experiment was performed. The ligand growing experiment started from a starter ligand, diethyl 6,8-dibenzyloxy-3,4-dihydroisoquinoline-3,3-dicarboxylate, which was then grown against a reference molecule, bestatin, using Spark 10.5.6 [24,25] by mapping a different region of the same active site. The fragment growing experiment with Spark identified viable replacements for the selected portion of a reference compound by using a series of fragment databases. In this experiment, molecular field technology was used, which condensed the molecular fields to a set of points around a molecule, termed as field points. 

**Figure 2:**
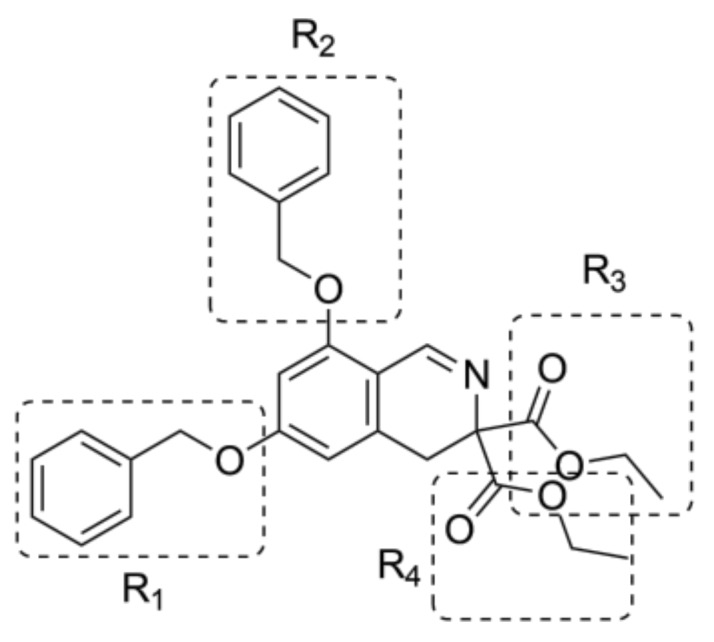
Chemical structure of the starter ligand, diethyl 6,8-dibenzyloxy-3,4-dihydroiso- quinoline-3,3-dicarboxylate, with selected substituents for replacement.

Each of four starter’s substituents, namely R_1_, R_2_, R_3_, and R_4_ (Figure 2), was selected for replacement, and a library containing 500 new derivatives was generated each time. Only one substituent (e.g., R_1_) was replaced each time, and the other substituents (e.g., R_2_, R_3_, and R_4_) remained unchanged. Finally, 2000 derivatives were obtained, which were filtered through Lipinski’s “rule of five” [22,23]. After filtration, a group of 485 compounds with no violation of Lipinski’s “rule of five” was chosen for further studies.

### 2.3. Molecular Docking

Virtual molecular docking is a computer-aided technique used for inexpensive and rapid identification of small compounds that bind to specific targets [26]. Virtual docking involves the docking of large libraries of compounds in the binding site of particular targets, thus potential ligands with potential binding affinity against the target can be selected for biological testing. Because the virtual docking method plays a key role in the identification of new compounds for the inhibition of protein targets, this method was used to identify novel LAP inhibitors. In this study, molecular docking was performed using ICM-Pro 3.8-5 software (Molsoft LLC, San Diego, USA) [27,28]. The filtered compounds from the established virtual library, including 11,585 compounds from ZINC, 13,011 compounds from PubChem, and 485 compounds from Spark, were docked into the binding site of 3D crystal structure of blLAP in complex with l-leucinal (PDB code: 1LAN) [29,30]. The protein structure of blLAP was precisely described previously [16,17,18,29,30]. All the generated binding poses were manually inspected to ensure correct positioning within the binding pocket with respect to the interactions of the ligand moieties with the amino acid residues relevant to the inhibitory activity [31]. Residues such as Lys250, Lys262, Met270, Asn330, Ala333, Asp273, Arg336, Thr359, Leu360, Gly362, Ile421, Ala451, and Met454 play an important role in the interactions of LAP with inhibitors [32]. These residues were used as a filter to discard the incorrect poses derived from the docking. Moreover, the compounds were ranked by a docking score. Scoring function was implemented to predict the biological activity by examining the interactions between the compound and potential target [18].

Docking of compounds from the ZINC library to 1LAN resulted in the selection of top 100 compounds with the best score; after duplicate deletion, only 68 compounds were chosen for further studies. The docking scores for the selected poses of the best compounds in the ZINC library were in the range of −25.18 to −51.09. Docking of compounds from the PubChem library resulted in the selection of 100 compounds with docking scores in the range from −28.07 to −51.72. 

**Figure 3:**
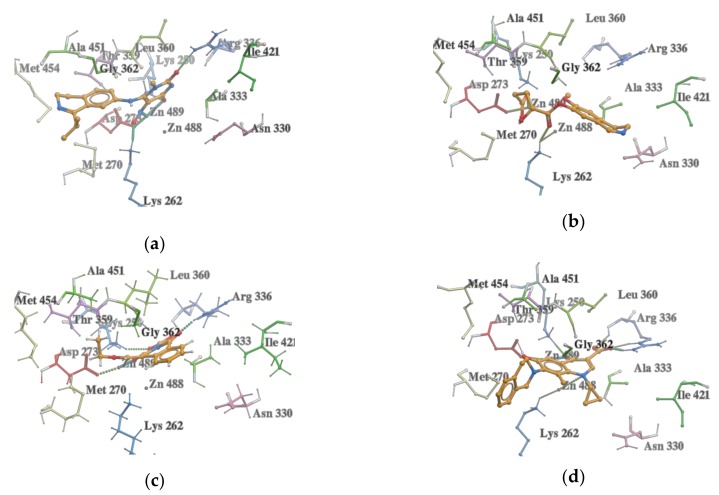
Interactions of the selected compounds with the best binding score values with amino acid residues and zinc ions in the leucine aminopeptidase (LAP) binding pocket. (**a**) 4-hydroxy-1-methyl-5-nitroso-6-{[1-(propan-2-yl)-3,4-dihydroisoquinolin-7-yl]amino}pyrimidin-2(1H)-one (score value of −51; ZINC database); (**b**) 4-hydroxy-5-[(6-hydroxy-3,4-dihydroisoquinolin-7-yl)oxy]-5-oxopentanoic acid (score value of −43.8; ZINC database); (**c**) 1,3-dioxo-4-(ethoxymethylene)-3,4-dihydroisoquinoline-2(1H)-carboxylic acid (score value of −51.72, PubChem); and (**d**) 1,4-dihydro-1-cyclopropyl-4-oxo-6-hydroxy-7-[3,4-dihydroisoquinoline-2(1H)-yl]-8-methylquinoline-3-carboxylic acid (score value of −42.93, PubChem).

All the compounds occupied the binding pocket of LAP and showed hydrogen bonds and hydrophobic interactions, which are typical for the inhibitory activity interactions with amino acid residues and zinc ions (Zn488, Zn489) in the active site (Figure 3). 

**Figure 4:**
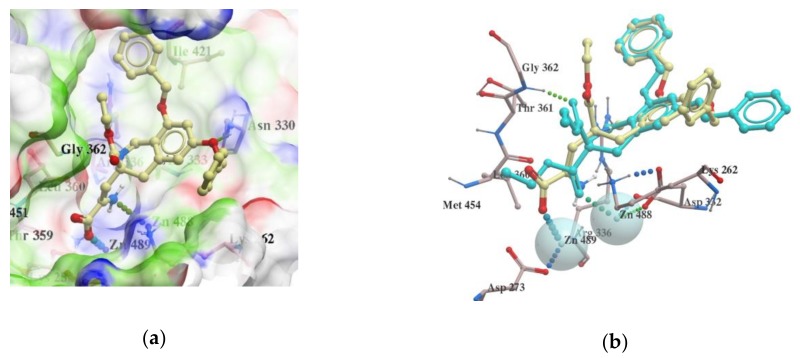
(**a**) interactions of LAP active site residues with the compound {[6,8-(dibenzyloxy)-3-(ethoxycarbonyl)-3,4-dihydroisoquinolin-3-yl]oxy}acetic acid with the best docking score (–38.66). (**b**) interactions of LAP active site residues with the compound {[6,8-(dibenzyloxy)-3-(ethoxycarbonyl)-3,4-dihydroisoquinolin-3-yl]oxy}acetic acid in comparison to docking pose of diethyl 6,8-dibenzyloxy-3,4,-dihydroisoquinoline-3,3-dicarboxylate (shown in cyan).

Compounds from Spark were also docked into the LAP active site, and the top 100 compounds with the best score in the range of −22.33 to −38.66 were then selected for 3D-QSAR using Forge software. The docking analysis indicated that the compound with the best docking score (−38.66) ({[6,8-(dibenzyloxy)-3-(ethoxycarbonyl)-3,4-dihydroisoquinolin-3-yl]oxy}acetic acid), shown in Figure 4, is placed in a similar way in the binding pocket of 1LAN as the starter ligand was docked in our previous studies [14,18].

### 2.4. VS with Lead Finder

**Figure 5:**
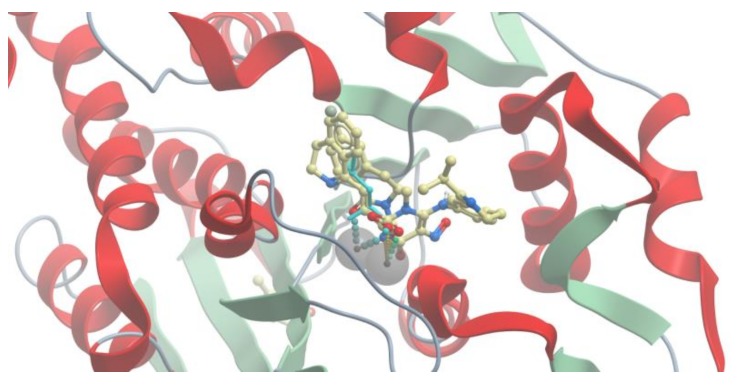
Compounds after docking with Lead Finder are placed similarly to the pose of leucine in the binding site of leucine aminopeptidase; leucine (cyan) is shown.

Subsequently, the top 268 highest ranked compounds (68 compounds from ZINC, 100 compounds from PubChem, 100 compounds from Spark) were subjected to docking using Lead Finder (LF) [33,34] and the predicted binding poses were compared with the relevant results obtained from ICM. The docking software LF has three specialized scoring functions designed to rank the predicted ligand poses, estimate the binding energy of the docked ligand poses, and rank compounds in VS experiments. The LF rank score values were in the range of −4.78 to −18.36 for all studied compounds (including rank score values between −7.71 and −16.39, −9.59 and −18.36, and −4.78 and −15.97 for compounds from ZINC, PubChem, and SPARK, respectively). The results of compounds’ docking with LF revealed that some ligands bound to LAP better than that found when docked with ICM. Docking using LF resulted in some cases of improved pose of the ligands in the binding pocket in comparison to ligand pose adopted after ICM docking. The ligands were bind similarly to l-leucinal in l-leucinal-blLAP complex (PDB code: 1LAN) (Figure 5).

### 2.5. 3D-QSAR

First, the 3D molecular structures of the dataset containing the selected 100 compounds from PubChem, 68 compounds from ZINC, and 100 compounds from SPARK were aligned to our previously published 3D-QSAR model for the LAN protein [18], and the compounds were then evaluated, using Forge software (10.6.0, Cresset^®^, Litlington, Cambridgeshire, UK) [25,35]. The leave-many-out (LMO) cross-validation was performed, by leaving out 20% of the molecules at each step for the same set of training and test set compounds as used in leave-one-out (LOO) cross-validation. The values of the LOO parameters, cross-validation correlation coefficient (q^2^), non-cross-validated correlation coefficient (r^2^), number of components (N), and predicted root mean square error (RMSE_pred_) were 0.717, 0.997, 4, and 0.661 respectively. The respective analogues values of the LMO parameters q^2^, r^2^, N, and RMSE_pred_ were 0.680, 0.997, 4, and 0.703, respectively. Similar values of parameters in both LOO and LMO cross-validation methods indicated the stability of the 3D-QSAR model. Statistical validation is a very important process for robust QSAR model development. The value of r^2^ should be >0.7 and the value of q^2^ should be >0.5 in the QSAR model for high predictive accuracy. This also confirmed that a reliable QSAR model was obtained in the present study.

In the entire library of 264 derivatives (after duplicates removal), the model provided excellent and good description for 35 and 51 compounds, respectively. This implies that most of the features of the evaluated molecules were well described by the training set of the 3D-QSAR model, and the predicted activity could be considered reliable. Other values, described as “bad” or “poor”, indicate that the molecule has field points that are not specified by the equation, resulting in unreliable predicted activities. The chemical structures and score values of all the compounds with excellent description by the model are presented in Supplementary Materials Table S1. The compounds with excellent description by the 3D-QSAR were predicted to possess pIC_50_ (pIC_50_ = −log (IC_50_)) activity between 3.8 and 5.7. A linear regression plot of experimental versus calculated pIC_50_ values is presented in Supplementary Materials Figure S4.

**Figure 6:**
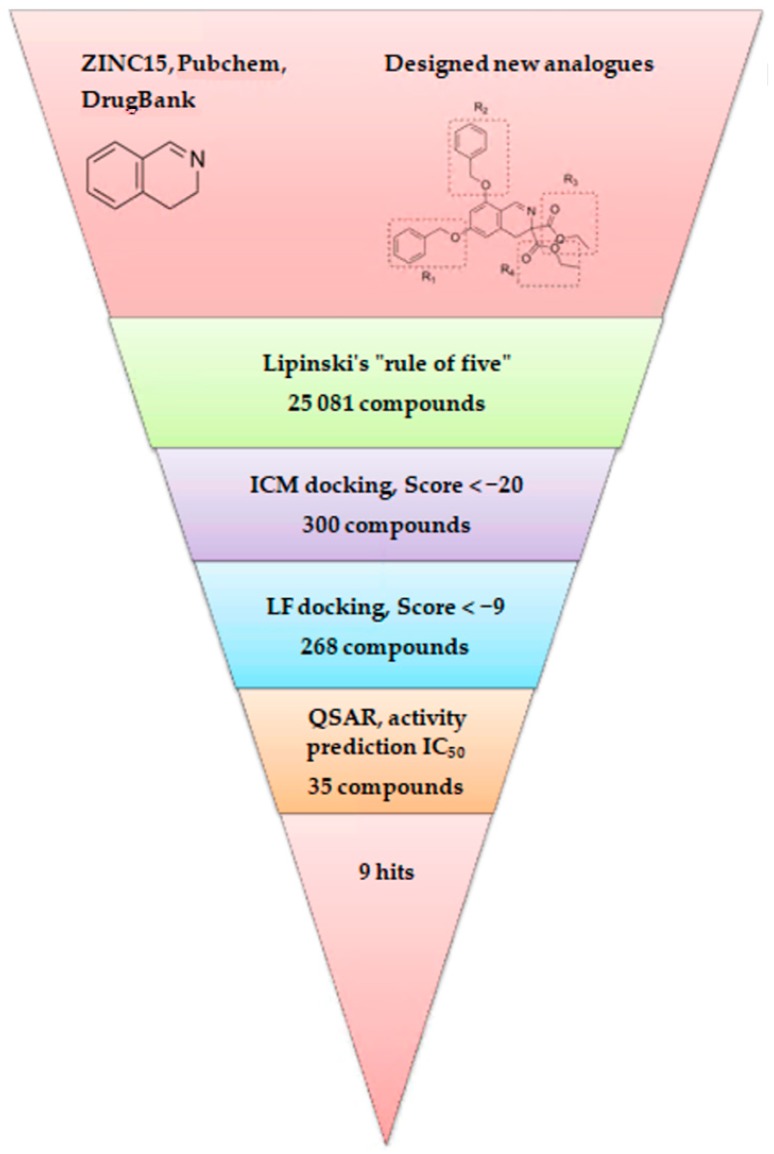
Schematic representation of the used workflow.

From 35 compounds with the excellent description by the 3D-QSAR model, 9 hit compounds were selected, which, in addition had the best ICM docking score values, below −32. The score value below −32 is regarded as a good indicator in compound selection by using ICM software [27,28]. After docking using LF and visual inspection, an improvement in ligand binding to LAP protein was observed but score values were not good indicators for ligand selection in that case. Thus, we decided to select ligands based on ICM docking score and results from 3D-QSAR. The chemical structures, pIC_50_ and ICM and LF score values of compounds one to nine are presented in Table 1. Schematic diagram of the underlying workflow of the LAP inhibitors drug discovery is shown in Figure 6.

### 2.6. In Silico Drug-Likeness, Bioavailability, and Toxicology Prediction

Drug-likeness studies measure the probability of a molecule to act as an oral drug in terms of its bioavailability [36,37]. As the interaction of an inhibitor with an enzyme cannot ensure its suitability as a drug, to further strengthen the results of 3D-QSAR and docking studies, we also performed in silico absorption, distribution, metabolism, and excretion (ADME) studies by using the SwissADME web tool [36]. The prediction of these properties is very essential in the drug design process and poor ADMET properties often account for the failure of approximately 60% of new chemical entities in clinical trials. Hence, SwissADME is a very valuable and reliable tool. In silico techniques for the prediction of ADMET properties are more attractive than conventional experimental assays because of the large numbers of compounds (both real and designed) that can be tested and because of significant time and cost reduction.

**Figure 7:**
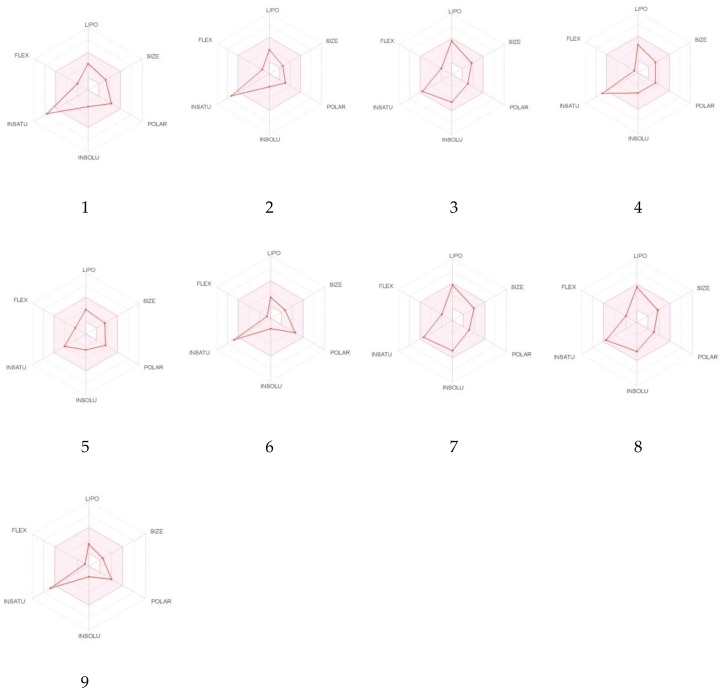
Radar plots of compounds 1–9. The pink area is a suitable physicochemical space for oral bioavailability. Lipophilicity (LIPO): −0.7 < XLOGP3 < 5.0; SIZE: 150 g/mol < MW < 500 g/mol; polarity (POLAR): 20 Å^2^ < topological polar surface area (TPSA) < 130 Å^2^; and insolubility (INSOLU): 0 < LogS < 6; INSATU (insaturation): 0.25 < fraction of carbons in sp^3^ hybridization < 1; FLEX (flexibility): 0 < number of rotatable bonds < 9. The radar plots were obtained using the SwissADME web tool [36].

**Figure 8:**
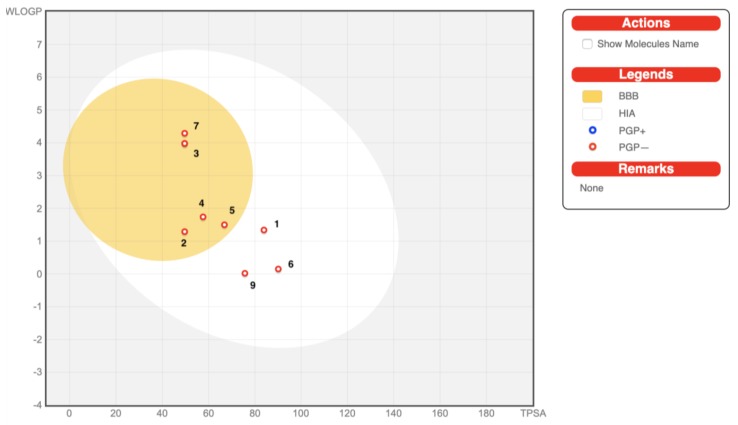
BOILED-Egg plot of compounds 1–9 with excellent description of the three-dimensional quantitative structure-activity relationship (3D-QSAR) model and the best ICM score values. Points located in the BOILED-Egg yolk (yellow) represent molecules predicted to passively permeate through the blood-brain barrier (BBB), whereas points in the egg white are predicted to be passively absorbed by the gastrointestinal tract (HIA). Blue dots (PGP+) indicate the molecules expected to be effluated from the central nervous system (CNS) by P-glycoprotein, whereas the red ones (PGP-) indicate the molecules predicted not to be effluated from the CNS by P-glycoprotein.

The oral bioavailability of compounds one to nine is shown in bioavailability radar plots (Figure 7). Compounds three, five, seven, and eight were predicted to be orally bioavailable. The other compounds (one, two, four, six, and nine) showed one off-shoot relative to unsaturation (INSATU), which implies that they could have suboptimal physicochemical properties for their oral bioavailability. Gastrointestinal (GI) absorption and blood-brain barrier (BBB) penetration which are relative to the absorption and distribution parameters are graphically presented in Figure 8. All the compounds were predicted to be passively absorbed by the GI tract. Compounds two to five and seven to nine showed BBB permeation, whereas the other compounds did not. None of the compounds were expected to be effluated from the central nervous system (CNS) by P-glycoprotein.

Metabolism plays an important role in the bioavailability of the drugs and drug–drug interactions. Cytochrome P-450 enzymes (CYPs) constitute the most significant class of enzymes for drug-likeness assessment. These enzymes are the site of the majority of drug–drug interactions. Ten human CYPs from seven subfamilies, namely CYP1A2, CYP2A6, CYP2B6, CYP2C8, CYP2C9, CYP2C19, CYP2D6, CYP2E1, CYP3A4, and CYP3A5 are responsible for the metabolism of most drugs [38]. Most of the compounds were found to be substrates of CYP1A2, except compounds one, five, six, and nine. Compounds seven and eight were substrates of CYP2C19. Compounds three and eight were substrates of CYP2C9 and CYP2D6. None of the compounds were found to be the substrate of CYP3A4 (Table 2).

Skin permeability coefficient (K_p_) was also predicted for the selected compounds. The K_p_ values were in the range of –5.19 to –7.27. The more negative the log K_p_ (with K_p_ in cm/s), the less skin permeant is the molecule [36,37]. The bioavailability score value of 0.56 (56%) for most of the compounds and 0.55 (55%) for molecule nine indicates the probability of their bioavailability, and it is based on total charge of compound, topological polar surface area (TPSA), and violation of Lipinski filter (Table 2).

Pan-assay interference compounds (PAINS) and Brenk filters were implemented to provide information regarding potentially problematic fragments (toxic, metabolically unstable, or possessing properties responsible for poor pharmacokinetics), in the chemical structures of compounds one to nine. Both filters showed alerts for compound six, while Brenk also showed alerts for compounds one and two [36] (Table 2).

Lead-likeness of the studied compounds was also calculated. Leads are molecules, which are subjected to chemical modifications, that will most likely increase size and lipophilicity. Thus, leads should be smaller and less hydrophobic than drug-like molecules [36]. Teague et al. [39] suggested that molecules with MW in the range of 100 to 350 Da and calculated logP in the range of 1 to 3 are greatly superior to those considered drug-like. In our study, compounds one, four, and five were shown to be lead-like (Table 2).

In addition to Lipinski′s “rule of five” [22,23], another four drug-likeness rules namely Ghose [40], Veber [41], Egan [42], and Muegge [43] were contemporarily satisfied for all the selected compounds.

The results of SwissADME calculations provide useful information about the selected compounds. Considering their GI absorption, metabolism through CYPs, and drug-likeness, all of them could be excellent candidates for further studies and manipulations. Moreover, the calculations results showed that compound one was predicted not only to be not metabolized by CYPs, not permeate through BBB and be passively absorbed by GI tract, but also it had superior properties than other compounds in context to its lead-likeness.

Predicted toxicity of compounds one to nine was calculated in admetSAR online tool [44,45]. The results of toxicity prediction are presented in Table 3. None of the compounds exhibited carcinogenicity and eye corrosion. Compounds three, five, seven, eight, and nine were predicted to not cause eye irritation. All of them, besides compound five, did not cause Ames mutagenesis. Compound five and six were determined to not be hepatotoxic. Most of the compounds belong to class III of acute oral toxicity, which means that according to US EPA classification, their LD_50_ values are greater than 500 mg/kg but less than 5000 mg/kg. Compounds two and nine belonged to category II of acute oral toxicity and their LD_50_ values are possibly greater than 50 mg/kg but less than 500 mg/kg.

These results provide essential information regarding toxicological profile of compounds one to nine and may be useful in selecting of route of administration and preferred dosage form. However, different values of probability indicated that these studies are preliminary and should be confirmed experimentally.

## 3. Materials and Methods

### 3.1. Database Search and Library Establishment

Different compound databases were searched for the availability of dihydroisoquinoline analogues. For this study, ZINC [19], PubChem [20], and DrugBank [21] were considered. The ZINC database substructure search of 3,4-dihydroisoquinoline reported 12,286 compounds (from 200,000,000 substances). After the removal of duplicates, 12,260 compounds were selected. Then, these compounds were filtered with parameters based on Lipinski’s “rule of five” [22,23], and finally a group of 11,585 compounds was obtained. The PubChem database (containing 102,628,457 compounds) provided 21,769 compounds and after consideration of Lipinski’s “rule of five”, 13,579 compounds were identified. A comparison of the obtained ZINC and PubChem sets of compounds revealed 600 duplicates, which were removed from the PubChem database, and after comparison with the ZINC database, only 13,011 were taken for further study. DrugBank database (including 13 491 compounds) did not reveal any significant hits. Finally, approximately 11,585 compounds from ZINC and 13,011 compounds from PubChem were considered for molecular docking and further in silico studies.

### 3.2. Ligand Growing Experiment

For the ligand growing experiment, Spark 10.5.6 software (Cresset^®^, Litlington, Cambridgeshire, UK) was used [24,25]. As a starter molecule, diethyl 6,8-dibenzyloxy-3,4,-dihydroisoquinoline-3,3-dicarboxylate in its bioactive conformation was chosen. Bestatin was loaded as a reference molecule to guide the ligand growth. The starter and reference molecules were pre-aligned in 3D. The score weight was set at 20% for the starter molecule, and at 80% for the reference molecule. A low score weight prevents large movements of the new molecule relative to the starter molecule. We then imported a protein, LAP (PDB code: 1LAN) to use it as an “excluded volume” around the starter molecule. During the Spark search, the excluded volume was checked to assess whether the replacement fragments clash with it. Four regions of the starter molecule were selected to be replaced (Figure 2). Each portion of the starter molecule was selected to be removed and replaced by the fragment from the databases. Ligand growing calculation method was used. Spark used three databases: ChEMBL_common (from literature reports) with 58,924 fragments and Commercial Very Common and Common (from commercially available compounds) with 20,561 and 42,778 fragments, respectively. Spark then searched these databases for any fragment with the correct number of connection points that geometrically match the broken bonds in the target. Matches were considered on the basis of both angle and distance. Fragments with the correct angles and distances between the connection points were merged into the target molecule to form the “product”. The latter was scored against the starter structure for electrostatic and shape similarity.

### 3.3. Molecular Docking

The crystal structure of blLAP complexed with l-leucinal (PDB code: 1LAN) was used for docking performed with ICM-Pro software (Molsoft LLC, San Diego, USA) [27,28,29]. All water molecules and co-crystallized ligands were removed from the PDB structure. Ligands were docked using a regular rigid receptor-flexible ligand docking approach that uses five potential energy maps. The maps were generated in a rectangular box with 0.5 Å grid spacing centered at the ligand binding site. Each molecule was first subjected to a conformational analysis outside of the protein pocket and a stack of low energy conformations is collected and used as starting geometries for grid docking. Ligand binding modes were scored according to the quality of the complex, and a user-defined number of the top scoring poses was re-ranked using the full ICM scoring function. The predicted score was calculated as the weighted sum of ligand-target van der Waals interactions and internal force field energy of the ligand, free energy changes due to conformational energy loss upon ligand binding, hydrogen bonding interactions, hydrogen bond donor-acceptor desolvation energy, solvation electrostatic energy upon ligand binding, hydrophobic free energy gain, and a size correction term proportional to the number of ligand atoms.

### 3.4. VS with Lead Finder

The full-atom model of LAP used in the current study was prepared from the PDB structure by adding hydrogen atoms and assigning ionization states of the amino acids with the Model Builder (*build_model*) program of the Lead Finder software package v 1.1.13 (BioMolTech^®^, Toronto, Ontario, Canada) [33,34]. VS of ligands to the prepared model of 1LAN and binding energy calculations were performed with Lead Finder v. 1.1.13 software using its default configuration parameters. The energy grid box for ligand docking was set at the geometrical center of the reference ligand to span 6 Å in each direction. Lead Finder assumes that the protein and ligand structures are rigid; however, it analyzes possible conformations of ligand structures by rotating functional groups along each freely rotatable bond (FRB). For each ligand pose Lead Finder determines values of the free energy of binding, the VS score, and, if applicable, the pose ranking score by using its three built-in scoring functions.

### 3.5. 3D-QSAR

All the optimized structures were imported into Forge software (10.6.0, Cresset^®^, Litlington, Cambridgeshire, UK) [35] for the evaluation of the dataset in the field-based 3D-QSAR model that was previously published [18]. All the molecules were aligned with the training set of the 3D-QSAR model. The negative, positive, shape, and hydrophobic field points of each molecule were generated using the extended electron distribution (XED) force field in Forge. Partial least squares (PLS) regression analysis was used to confirm the predictive ability of the field-based 3D-QSAR model. Statistical parameters, such as non-cross-validated correlation coefficient (r^2^), cross-validated correlation coefficient (q^2^), and RMSE_pred_ were calculated. The maximum number of components (N) was fixed to 20, while the maximum distance for sample point was set to 1Å. The LOO and LMO cross-validation methods were used. The parameters used in the conformation search, alignment, and model building are reported in Supplementary Materials Figures S1–S3.

### 3.6. In Silico Drug-Likeness, Bioavailability, and Toxicology Prediction

The SwissADME web tool was used to evaluate compounds’ ADME [36]. It was accessed at http://www.swissadme.ch. Two dimensional (2D) chemical structures of the selected compounds were drawn and transferred as a list of molecules, defined by canonical simplified molecular input line entry specification (SMILES). All descriptors and molecular parameters were computed through the OpenBabel API (version 2.3.0, 2012). The pharmacokinetics section proposed one linear method for skin permeation, which relied on the quantitative structure-property relationship (QSPR) model developed by Potts and Guy that links the decimal logarithm of K_p_ with MW and logP [36,37]. Multiple linear regression was used to predict K_p_. The prediction for passive human GI absorption (HIA) and BBB penetration consisted of the readout of the BOILED-Egg model, an intuitive graphical classification model. 

Lipinski’s “rule of five” (Pfizer) [22,23], Ghose (Amgen) [40], Veber (GSK) [41], Egan (Pharmacia) [42], and Muegge (Bayer) [43] rules were used for drug-likeness pre-screening studies.

For prediction of toxicological profile of selected compounds, we used admetSAR (version 2.0) online tool. It can be accessed at http://lmmd.ecust.edu.cn/admetsar2 [44,45]. Models used in the study were described in detail on the mentioned source file.

## 4. Conclusions

The cumulative risk of cancer incidence indicates that 1 in 8 men and 1 in 10 women will develop cancer in their lifetime [2]. When coupled with the estimated cost of US $1.16 trillion per year for cancer treatment and care, this clearly makes cancer a public health priority [1]. Despite significant progress in anti-cancer therapies, some cancers continue to have poor prognosis, which necessitates the development of new chemical entities. Considering that the process of introducing a new drug in the market is very difficult and time and money consuming, pharmaceutical companies and researchers pay much attention to computer-aided drug discovery techniques in various stages of drug discovery and development to minimize the failure rate [9]. Despite some disadvantages of in silico drug design methods such as inaccuracy of scoring functions for the evaluation of target-ligand binding free energy, difficulty of considering target flexibility in docking, or high false positive rate of VS [8,46], these methods have already helped to successfully introduce in market drug molecules, such as imatinib and other tyrosine kinase inhibitors [47], which have revolutionized leukemia treatment and enabled to treat cancer as a chronic disease like diabetes [48]. 

In the present study, the screening of new potential LAP inhibitors with a scaffold based on 3,4-dihydroisoquinoline from two databases: ZINC and PubChem as well as drug design using Spark software was performed. Through a statistical/computational filtration approach based on 2D descriptors, docking calculations, and 3D-QSAR statistical models, several and potentially active 3,4-dihydroisoquinoline derivatives were obtained. Finally, nine selected compounds with good drug-like, ADME pharmacokinetics parameters and toxicological profile were provided. Especially, compound one occurred to be a valuable starting point for the design of new synthetic derivatives with improved activity. The next steps will include the chemical synthesis of selected compounds, *in vitro* studies to experimentally confirm their inhibitory activity towards LAP and antiproliferative activity on cancer cell lines. In conclusion, these studies indicated that compounds with 3,4-dihydroisoquinoline moiety have potential to inhibit LAP and further studies on this topic are needed. 

## Figures and Tables

**Table 1:** Chemical structures and calculated pIC_50_ values of the selected compounds.

Compound’s number/ID	Chemical Name	Chemical Structure	pIC_50_	ICM Score	LF Score
1/ PUBCHEM 101710591	1,3-dioxo-4-(ethoxymethylene)-3,4-dihydroisoquinoline-2(1*H*)-carboxylic acid	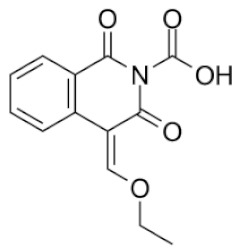	4.8	−51.73	−12.63
2/ ZINC 238690488	(*E*)-3-(3,4-dihydroisoquinolin-1-yl)acrylic acid	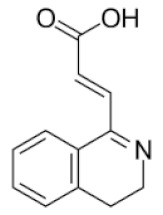	4.6	−40.09	−10.63
3/ ZINC 1243196903	5-(1-isopropyl-3,4-dihydroisoquinolin-7-yl)-2-methylbenzoic acid	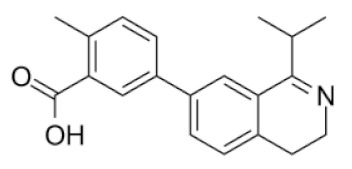	4.3	−35.99	−10.23
4/ PUBCHEM 67293279	6-bromo-1-oxo-3,4-dihydroisoquinoline-2-carboxylic acid	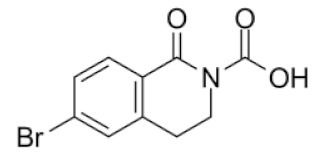	4.9	−35.37	−12.97
5/ PUBCHEM 82579683	3-methyl-1-oxo-2-(oxolan-2-ylmethyl)-3,4-dihydroisoquinoline-4-carboxylic acid	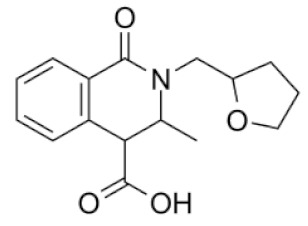	4.5	−34.91	−10.80
6/ PUBCHEM 135927986	(3S)-6,7-dihydroxy-3,4-dihydroisoquinoline-3-carboxylic acid	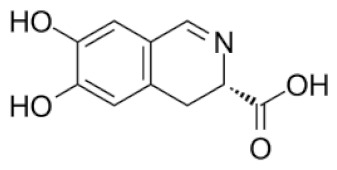	4.8	−33.99	−11.39
7/ ZINC 1206051829	2,3-difluoro-5-(1-isopropyl-3,4-dihydroisoquinolin-7-yl)benzoic acid	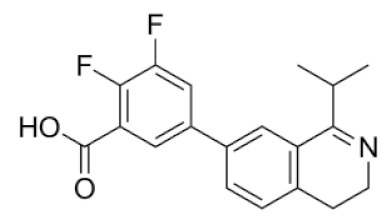	4.6	−33.92	−11.06
8/ ZINC 1243180093	3-(1-isopropyl-3,4-dihydroisoquinolin-7-yl)-5-methylbenzoic acid	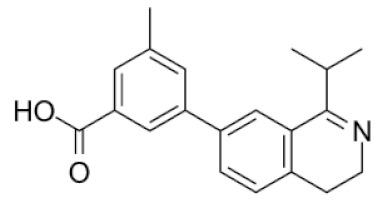	4.5	−32.84	−12.23
9/ ZINC 34115917	(3S)-1-amino-3,4-dihydroisoquinoline-3-carboxylic acid	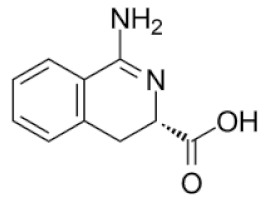	4.4	−32.12	−10.73

**Table 2:** Pharmacokinetic and medicinal chemistry parameters of compounds 1–9.

Pharmacokinetic Parameters	Compounds								
	1	2	3	4	5	6	7	8	9
GI Absorption ^a^	high	high	high	high	high	high	high	high	high
BBB ^b^ Barrier Permeation	no	yes	yes	yes	yes	no	yes	yes	no
PGP ^c^ Substrate	no	no	no	no	no	no	no	no	no
CYP ^d^ 1A2 Inhibitor	no	yes	yes	yes	no	no	yes	yes	no
CYP ^d^ 2C19 Inhibitor	no	no	no	no	no	no	yes	yes	no
CYP ^d^ 2C9 Inhibitor	no	no	yes	no	no	no	no	yes	no
CYP ^d^ 2D6 Inhibitor	no	no	yes	no	no	no	no	yes	no
CYP ^d^ 3A4 Inhibitor	no	no	no	no	no	no	no	no	no
Log Kp (Skin Permeation ^e^)	−6.57	−6.53	−5.19	−6.16	−7.01	−7.27	−5.44	−5.19	−7.08
Leadlikeness	yes	no	no	yes	yes	no	no	no	no
Bioavailability Score	0.56	0.56	0.56	0.56	0.56	0.56	0.56	0.56	0.55
PAINSBrenk	0	0	0	0	0	1	0	0	0
	3	1	0	0	0	1	0	0	0

^a^ Gastrointestinal absorption; ^b^ Blood-brain barrier penetration; ^c^ P-glycoprotein substrate; ^d^ Cytochrome P450; ^e^ cm/s.

**Table 3:** Toxicity prediction for compounds 1–9.

Compounds	1	2	3	4	5	6	7	8	9
Carcinogenicity	−(0.73)	−(0.83)	−(0.89)	−(0.84)	−(0.81)	−(0.76)	−(0.86)	−(0.89)	−(0.87)
Eye corrosion	−(0.98)	−(0.95)	−(0.98)	−(0.98)	−(0.99)	−(0.99)	−(0.98)	−(0.98)	−(0.99)
Eye irritation	+(0.85)	+(0.94)	−(0.96)	+(0.85)	−(0.96)	+(0.86)	−(0.98)	−(0.92)	−(0.79)
Ames mutagenesis	−(0.61)	−(0.82)	−(0.65)	−(0.74)	+(0.51)	−(0.75)	−(0.63)	−(0.74)	−(0.66)
Hepatotoxicity	+(0.63)	+(0.73)	+(0.83)	+(0.55)	−(0.55)	−(0.53)	+(0.83)	+(0.80)	+(0.53)
Acute oral toxicity	III (0.63)	II (0.42)	III (0.51)	III (0.60)	III (0.73)	III (0.41)	III (0.53)	III (0.53)	II (0.37)

“+” means toxic; “−“means nontoxic. The numbers in brackets indicate the probability.

## Supplementary Materials

The following are available online, Figure S1: Forge’s parameters used for the conformation hunt, Figure S2: Forge’s parameters used for the alignment, Figure S3: Forge’s parameters used for building a model, Figure S4: Linear regression plot of experimental versus calculated pIC_50_ values used in 3D-QSAR model, Table S1: Chemical structures, pIC_50_, ICM and LF score values for the compounds with an excellent description by the model.
