# Supplementary material for: In Silico Screening for Novel Leucine Aminopeptidase Inhibitors with 3,4-Dihydroisoquinoline Scaffold
Source: Molecules. 2020 Apr 10;25(7):1753. doi: 10.3390/molecules25071753 (PMC7180978; doi:10.3390/molecules25071753)
Supplement: Supplementary file 1 [file molecules-25-01753-s001.pdf]

Supplementary material

In silico screening for novel leucine aminopeptidase inhibitors with 3,4-dihydroisoquinoline scaffold

Joanna Ziemska [1*], Jolanta Solecka [1] and Małgorzata Jarończyk [2]

[1] National Institute of Public Health – National Institute of Hygiene, Chocimska 24, Warsaw 00-791, Poland; jziemska@pzh.gov.pl

[2] National Medicines Institute, Chełmska 30/34, Warsaw 00-725; m.jaronczyk@nil.gov.pl

* Correspondence: jziemska@pzh.gov.pl; tel.: +48 22 54 21 319

Table of content

Fig. S1 Forge's parameters used for the conformation hunt
Fig. S2 Forge's parameters used for the alignment
Fig. S3 Forge's parameters used for building a model
Fig. S4 Linear regression plot of experimental versus calculated pIC_{50} values used in 3D-QSAR model
Table S1. Chemical structures, pIC_{50}, ICM and LF score values for the compounds with an excellent description by the model

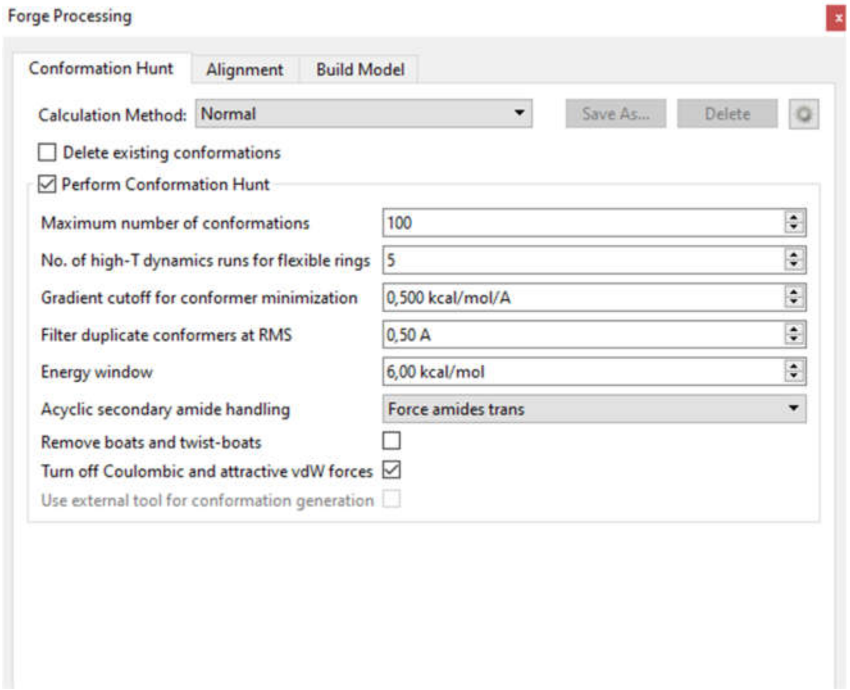

Fig. S1 Forge's parameters used for the conformation hunt

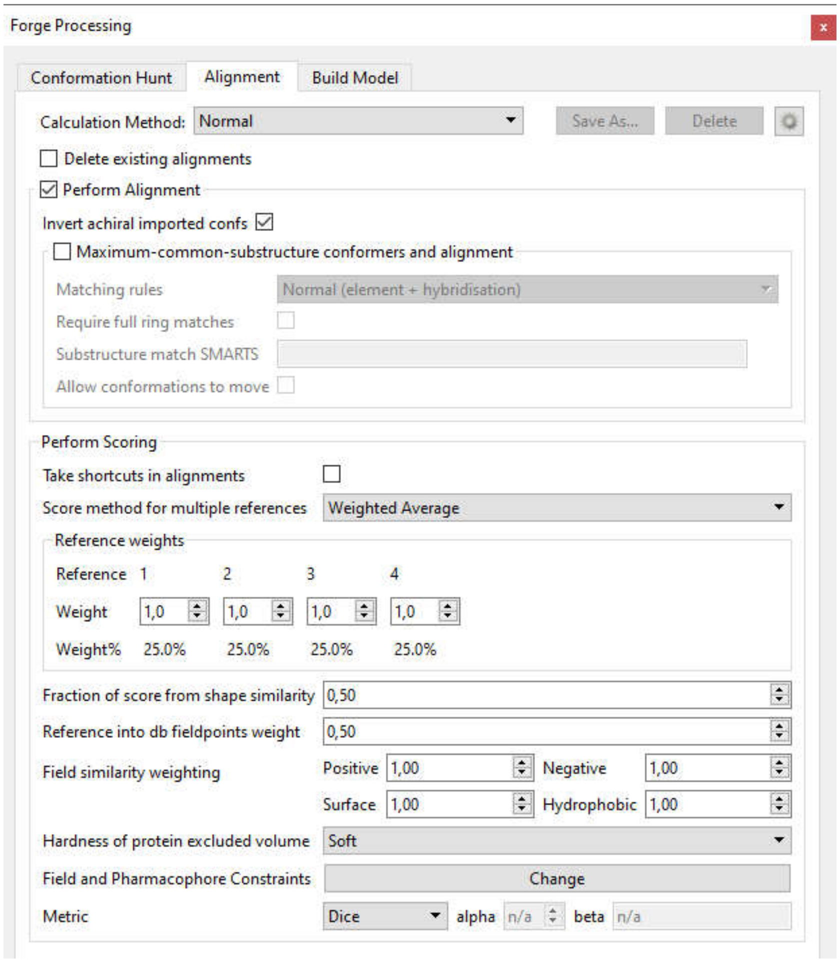

Fig. S2 Forge's parameters used for the alignment

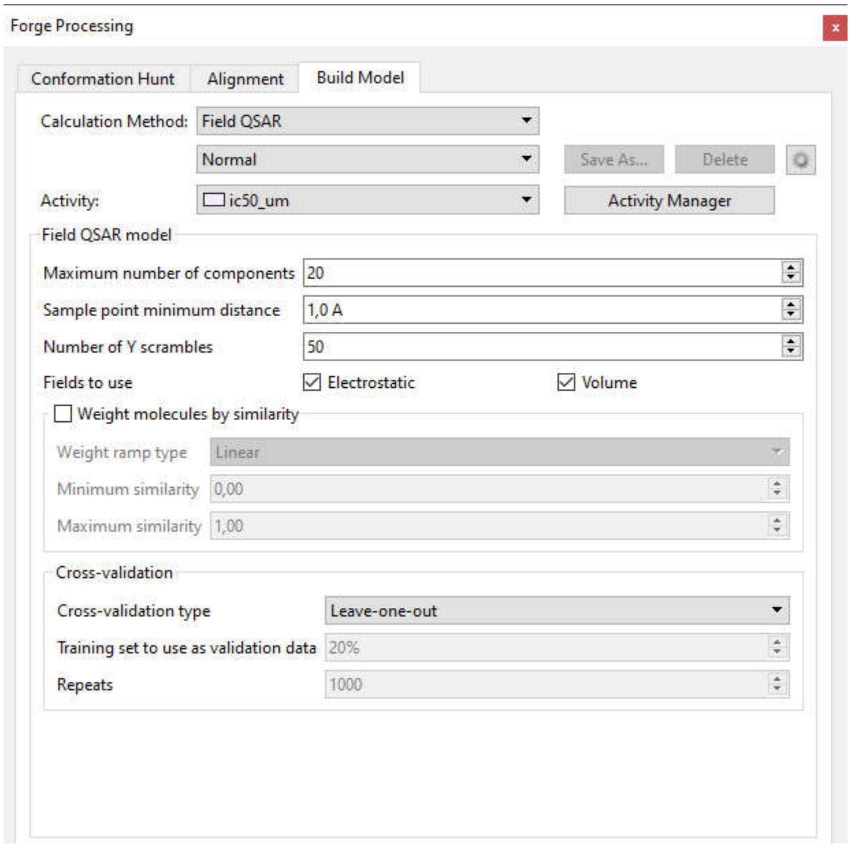

Fig. S3 Forge's parameters used for building a model

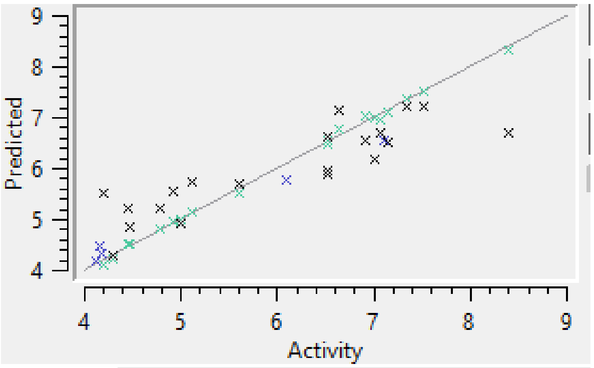

Fig. S4 Linear regression plot of experimental versus calculated (predicted) pIC_{50} values used in 3D-QSAR model

Table S1. Chemical structures, pIC_{50}, ICM and LF score values for the compounds with an excellent description by the model

Compound number	SMILES	pIC_{50}	ICM score	LF score
PUBCHEM 101710591	CCOC=C1C2=CC=CC=C2C(=O)N(C1=O)C(=O)O	4.8	-51.73	-12.63
ZINC 238690488	OC(=O)\C=C\C1=NCCC2=CC=CC=C12	4.6	-40.09	-10.63
ZINC 1243196903	Cc1ccc(-c2ccc3c(c2)C(C(C)C)=NCC3)cc1C(=O)O	4.3	-35.99	-10.23
PUBCHEM 67293279	OC(=O)N1CCC2=C(C=CC(Br)=C2)C1=O	4.9	-35.37	-12.97
PUBCHEM 82579683	CC1C(C(O)=O)C2=CC=CC=C2C(=O)N1CC1CCCO1	4.5	-34.91	-10.80
PUBCHEM 135927986	C1[C@H](N=CC2=CC(=C(C=C21)O)O)C(=O)O	4.8	-33.99	-11.39
ZINC 1206051829	CC(C)C1=NCCC2=C1C=C(C=C2)C1=CC(C(O)=O)=C(F)C(F)=C1	4.6	-33.92	-11.06
ZINC 1243180093	Cc3cc(C(=O)O)cc(c2ccc1CCN=C(C(C)C)c1c2)c3	4.5	-32.84	-12.23
ZINC 34115917	NC1=NC(CC2=C1C=CC=C2)C(O)=O	4.4	-32.12	-10.73
PUBCHEM 117846521	OC(=O)C1CC2=C(C=CC=C2)C=N1	5.0	-31.84	-10.99
PUBCHEM 16451703	OC(=O)C1CC2=CC=CC=C2C(=O)N1CCC1=CC=CC=C1	4.4	-31.58	-13.32
ZINC 1240849935	CC(C)C1=NCCC2=C1C=C(C=C2)C1=CC=C(S1)C(O)=O	4.7	-30.71	-10.64
PUBCHEM 84643346	CN1C(CC2=C(C=C(Br)C=C2)C1=O)C(O)=O	3.8	-30.60	-11.24
PUBCHEM 84629545	CN1C(CC2=C(C=C(Cl)C=C2)C1=O)C(O)=O	3.8	-30.55	-11.11
PUBCHEM 7464895	OC(=O)C1CC2=CC=CC=C2C(=O)N1CCC1=CC=CC=C1	3.9	-30.44	-13.31
PUBCHEM 82579602	CN1C([C@@H](C(O)=O)C2=C(C=CC=C2)C1=O)C1=CC=CN1	4.8	-30.30	-12.25
PUBCHEM 101490190	NC1=[N](O)C(CC2=CC=CC=C12)[C@H](O)C1=CC=CC=C1	4.9	-30.10	-18.36
PUBCHEM 10584362	COC1=C2C=NC(CC2=CC=C1)C(O)=O	4.3	-30.02	-10.02

ZINC 329852746	C\N=C(\O)C1(CC1)C(\O)=N/CC1=CC2=C(CCN=C2O)C=C1	4.5	-29.65	-12.16
PUBCHEM 94217874	C1CC(OC1)CN2C(C(C3=CC=CC=C3C2=O)C(=O)O)C4=CC=CC=C4	5.3	-29.55	-15.05
PUBCHEM 10706917	C1C(N(C(=O)C2=CC=CC=C21)C3=CC=CC=C3)C(=O)O	4.7	-29.52	-12.58
PUBCHEM 82579659	CC(C)CN1C(C(C2=CC=CC=C2C1=O)C(=O)O)C3=CC=CC=N3	5.2	-29.49	-12.87
PUBCHEM 84623274	CC1=CC2=C(CC(N(C2=O)C)C(=O)O)C=C1	4.2	-29.36	-10.89
PUBCHEM 84627455	CCC1=CC2=C(CC(N(C2=O)C)C(=O)O)C=C1	3.9	-29.24	-10.56
PUBCHEM 92975769	C1C(N=C(C2=CC=CC=C21)N)C(=O)O	4.8	-29.20	-10.54
PUBCHEM84624333	CN1C(CC2=C(C1=O)C=C(C=C2)F)C(=O)O	3.8	-29.20	-10.54
PUBCHEM 82579634	C1CC1N2C(C(C3=CC=CC=C3C2=O)C(=O)O)C4=CC=CC=N4	5.7	-28.87	-13.16
PUBCHEM 10035418	C1C(N=C(C2=CC=CC=C21)N)C(=O)O	4.7	-28.86	-9.82
PUBCHEM 22018502	COC1=CC2=C(C=C1)C(=C)N(CC2)C(=O)O	4.6	-28.85	-9.91
PUBCHEM 102587128	CC1=CC(=CC(=C1)N2C(C(C3=CC=CC=C3C2=O)C(=O)O)C4=CC=CC=C4)C	4.8	-28.18	-14.70
PUBCHEM 22334717	COCCN1C(C(C2=CC=CC=C2C1=O)C(=O)O)C3=CC=CS3	5.1	-28.10	-12.28
ZINC 1205610162	CC(C)C1=NCCc2ccc(-c3ccsc3C(=O)O)cc21	4.8	-27.70	-12.48
ZINC 205972081	OC(=O)C1=CC=C(C=C1)C1=NCCC2=CC=CC=C12	4.1	-25.69	-12.17
ZINC 575441179	OC(=O)C1CC2=CC(O)=C(O)C(O)=C2C=N1	5.3	-25.33	-11.09
SPARK	CCOC(=O)C1(CC2=C(C=N1)C(OCC1=CC=CC=C1)=CC(=C2)C(=C)C1=NNC=C1N)C(=O)OC	4.6	-22.63	not determined

20 by the authors. Submitted for possible open access publication under the terms and conditions of the Creative Commons Attribution (CC BY) license (http://creativecommons.org/licenses/by/4.0/).
